# The effects of life stress and neural learning signals on fluid intelligence

**DOI:** 10.1007/s00406-014-0519-3

**Published:** 2014-08-21

**Authors:** Eva Friedel, Florian Schlagenhauf, Anne Beck, Raymond J. Dolan, Quentin J.M. Huys, Michael A. Rapp, Andreas Heinz

**Affiliations:** 1 Department of Psychiatry and Psychotherapy, Charité – Universitätsmedizin Berlin, Campus Charité Mitte, Charitéplatz 1, 10117 Berlin, Germany; 2 Max Planck Institute for Human Cognitive and Brain Sciences, Leipzig, Germany; 3 Gatsby Computational Neuroscience Unit, University College London, London, UK; 4 Translational Neuromodeling Unit, Institute for Biomedical Engineering, University of Zurich and ETH Zurich, Zurich, Switzerland; 5 Department of Psychiatry, Psychotherapy and Psychosomatics, University Hospital of Psychiatry, Zurich, Switzerland; 6 Social and Preventive Medicine, University of Potsdam, Potsdam, Germany; 7 Cluster of Excellence NeuroCure, Charite-Universitätsmedizin Berlin, Berlin, Germany

**Keywords:** Reinforcement learning, Prediction error signal, Ventral striatum, Stress, Intelligence

## Abstract

Fluid intelligence (fluid IQ), defined as the capacity for rapid problem solving and behavioral adaptation, is known to be modulated by learning and experience. Both stressful life events (SLES) and neural correlates of learning [specifically, a key mediator of adaptive learning in the brain, namely the ventral striatal representation of prediction errors (PE)] have been shown to be associated with individual differences in fluid IQ. Here, we examine the interaction between adaptive learning signals (using a well-characterized probabilistic reversal learning task in combination with fMRI) and SLES on fluid IQ measures. We find that the correlation between ventral striatal BOLD PE and fluid IQ, which we have previously reported, is quantitatively modulated by the amount of reported SLES. Thus, after experiencing adversity, basic neuronal learning signatures appear to align more closely with a general measure of flexible learning (fluid IQ), a finding complementing studies on the effects of acute stress on learning. The results suggest that an understanding of the neurobiological correlates of trait variables like fluid IQ needs to take socioemotional influences such as chronic stress into account.

**Electronic supplementary material:**

The online version of this article (doi:10.1007/s00406-014-0519-3) contains supplementary material, which is available to authorized users.

## Introduction

Fluid intelligence (fluid IQ) [1, 2] describes the capacity for rapid problem solving and flexible adjustment to an ever-changing environment. Its expression is a general factor comprising attributes such as attention, cognitive speed, working memory, reasoning, and episodic memory that have manifold impacts on learning. One important neurobiological correlate of learning is the phasic activation of dopamine neurons in the VTA [3–5] which is reflected in BOLD fMRI studies in humans as a prediction error (PE) in a number of regions including the ventral striatum (VS) [6]. The prominent place of the VS PE in neurobiological accounts of learning derives from the fact that it is a particular type of teaching signal, which indicates the need for a change in expectation, as well as the direction and quantity of change necessary to acquire habits [7–9]. Animal experiments have shown that PE signaling is associated with phasic dopamine firing with the size of PE reflecting the amount of dopamine release [4, 10]. In humans, PE signaling has also been shown to phasically release striatal dopamine (indirectly measured via displacement of dopamine D2 receptor ligands) [11] and to be modulated by more tonic aspects of dopamine synthesis capacity measured over 1 hour [12].

Recently, we reported that individual differences in fluid IQ are associated with VS BOLD PE signals, with stronger VS BOLD PE correlates in subjects with higher IQ [13]. At the same time, there is recent evidence that acute stress increases BOLD responses elicited by aversive PE signals in the VS [14]. Furthermore, fluid IQ and stress are well known to interact, with stress having a strong moderating influence on cognitive abilities [15, 16], reward learning [17, 18], risk taking [19], reward responsivity [20], and decision-making speed [19]. Also, stress due to social exclusion situations impairs cognitive speed and accuracy [21]. However, despite strong evidence for an impact of stress on cognition and decision-making processes, little is known about the specific patterns and moderating trait components underlying these changes on a behavioral and neuronal level. Interestingly, it has recently been observed that (on a behavioral level) acute stress does not impair so-called model-free reward learning, while more cognitively demanding model-based reward learning is more affected by acute stress when working memory capacity is lower [16].

With respect to dopaminergic transmission, a series of animal experiments indicates that acute as well as chronic stress moderated dopamine release and may thus interact with dopamine-dependent PE signaling in the striatum [22, 23]. For instance, changes in cortisol levels during an acute stressor were correlated with increases in striatal responses during a decision-making task [24]. Given these interactions between stress, dopaminergic PE signals, and aspects of fluid intelligence, we wanted to explore the effects of chronic stress on PE signaling and fluid IQ. One possibility is that stress increases dopamine release, which in turn increases PE signaling, and in accordance with our previous observation [12], fluid IQ. However, this is unlikely given the predominantly negative interactions between stress exposure and fluid IQ (a measure closely related to cognitive capacity and cognitive speed). We therefore tested the hypothesis that stress exposure modulates PE signaling above and beyond the previously observed correlation between PE signaling and fluid IQ.

To assess PE-related VS activity, we regressed VS BOLD signals onto PEs derived from a simple learning algorithm in which PEs are accumulated over time to form predictions. We note that this simple learning algorithm is ‘model-free,’ in that it only requires subjects to iteratively track what outcomes are observed after their choices, but it does not require subjects to have any explicit model or understanding of the task. We then asked whether this correlation, henceforth termed the VS BOLD PE signal, (1) is associated with measures of chronic life stress and (2) whether chronic life stress and VS BOLD PE signal alone or in interaction contribute to individual differences in fluid IQ.

## Materials and methods

### Subjects and screening instruments

A group of 16 right-handed healthy men with a mean age of 38.4 years (SD = 11.9; range 22–61*)* underwent fMRI and neuropsychological testing as a subgroup of a sample previously reported [13]. Subjects with Axis I and II psychiatric disorders according to DSM IV were excluded through the Structured Clinical Interview, and drug abuse was further excluded with urine tests.

### Neuropsychological assessment, intelligence measures, and stressful life events

A neuropsychological battery was administered within 2 months of fMRI measurements. Components of fluid and crystallized IQ were measured with an adaptation of the standard battery used in the Berlin Aging Study [25]. Fluid IQ was measured with a battery of nine tests comprising cognitive speed, attention and executive function, working memory, episodic memory, and reasoning. Cognitive speed was measured using the Digit Symbol Substitution test and the Reitan Trailmaking test, part A. Attention and executive function was measured using the Reitan Trailmaking test, part B [26], and Stroop [27] tests. Working memory was measured using forward and backward digit span tests [28]. Episodic memory was assessed using story recall with the German version of the Rivermead Behavioral Memory Test [29] and a German version of the auditory verbal learning test. Reasoning was measured using a test of figural analogies [30]. Fluid IQ was derived from a factorial analysis of the raw scores of each of these tests. Specifically, we used a Varimax rotation with an Eigenvalue cutoff set to 1.0; the final (single factor) solution accounted for 74.3 % of the variance in cognitive speed, attention, working memory, memory, and reasoning. Crystallized IQ was estimated using a verbal knowledge test, during which subjects are required to identify each single meaningful word from a total of 42 lists of five words.

Stressful life events (SLES) were assessed using the Life Events Scale [31], in which subjects were asked to self-report the presence of a stressful life event during the past 2 years from a list of 42 life events. We used the number of SLES as the main outcome measure.

### Reversal learning task

**Fig. 1:**
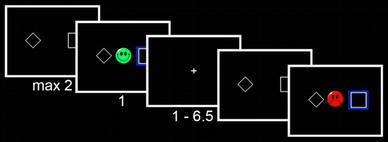
Probabilistic reversal task. Subjects first saw two abstract stimuli for up to 2 s (or reaction time). After selecting one with a button press, a blue frame surrounded the chosen target along with either positive (reward) or negative (loss) feedback. Feedback was displayed for 1 s, followed by a fixation cross for 1–6.5 s

During fMRI acquisition, subjects performed a reversal learning task (Fig. 1) known to evoke a BOLD PE signal in the striatum [13, 32]. In each of 200 trials (100 per session), subjects first saw two abstract targets on the screen and were asked to choose one of them as quickly as possible by pressing the left or right button with the left or right thumb on a response box (maximum response time: 2 s). A blue box surrounding their chosen target and feedback (either a green smiley face for positive feedback or a red frowning face for negative feedback) was simultaneously shown for 1 s. The trials were separated by a jittered interval of 1–6.5 s. Reward allocation was determined as follows: There were three types of reward allocation (i.e., block types): (1) 20 % for the left-hand response and 80 % for the right-hand response leading to reward, (2) 80 % for the left-hand response and 20 % for the right-hand response leading to reward, and (3) 50 % reward for the left-hand response and for the right-hand response. Block types changed unpredictably for the subject when two criteria were fulfilled: (1) minimum of 10 trials and (2) minimum of 70 % correct responses in the entire block. If subjects did not reach learning criteria after 16 trials, the task went over to the next block automatically.

### Computational modeling of reinforcement learning

The trial-by-trial sequence of choices for each subject was fit by a simple Rescorla-Wagner (RW) model in which behavior is driven by the expectation of rewards and in which trial-by-trial behavioral adaptation is proportional to the difference between the expected and obtained rewards [7]. More specifically, the model assumes that the likelihood of choosing action *a* on trial *t* is proportional to the reinforcement *Q*_*t*_(*a*) the subject expects to receive on that trial. The proportionality between the expected value and the choice probability is given by the softmax rule, which defines the probability *p*(*a*) of a certain action *a* as a function of the expected value, *Q*_*t*_, as follows:

$$p\left( {a|Q_{t} } \right) \, = \, \exp \left( {Q_{t} \left( a \right)} \right) \, / \, \left( {\varSigma_{{a^{\prime}}} \exp \left( {Q_{t} \left( {a^{\prime}} \right)} \right)} \right)$$

Note that this simply turns expectations of rewards for actions into action probabilities, ensuring that *p*(*a*) > *p*(*a*′) if and only if *Q*(*a*) > *Q*(*a*′) and that 0 ≤ *p*(*a*) ≤ 1. The expected value *Q*_*t*_(*a*_chosen_) in turn is updated iteratively:

$$Q_{t} \left( {a_{\text{chosen}} } \right) \, = \, Q_{t - 1} \left( {a_{\text{chosen}} } \right) \, + \varepsilon \left( {R_{t} {-} \, Q_{t - 1} \left( {a_{\text{chosen}} } \right)} \right)$$

where *ε* is the learning rate and *R*_*t*_ the reward obtained. The difference *R*_*t*_–*Q*_*t−*1_(a) is the reward PE. Thus, if *R*_*t*_ > *Q*_*t*_(*a*_chosen_), then *Q*_*t*_(*a*_chosen_) is increased, leading to a higher probability of being chosen on the next trial. Note that, here, the variable *R*_*t*_ represents the (effective) reinforcement sensitivity as expressed by the effect of the reinforcement on the subject’s choice behavior. This variable takes on value *R*_*t*_ = *β*_rew_ if a reward was obtained and *R*_*t*_ = −*β*_pun_ if a punishment was obtained. For each individual, a learning rate ε’ and the reinforcement sensitivity for reward and punishment (*β*_rew_′ and *β*_pun_′) were computed as the maximum a posteriori estimates of these parameters using a Gaussian prior.

Model fitting parameter estimation was performed in a hierarchical model with empirical priors treating parameters as a random effect. For an in-depth description, please compare [33, 34]. Briefly, prior to fitting the models, the learning rate was inverse sigmoid transformed and the reward sensitivities were log-transformed. This enforced the constraint that 0 ≤ ε ≤ 1 and that *β*_pun_ ≥ 0 and *β*_rew_ ≥ 0. Letting θ = [ε′, *β*_pun_′, *β*_rew_′] denote the vector of transformed parameters, we report the maximum a posteriori estimates of these parameters using a Gaussian prior with mean and variance parameters *μ* and *Σ*:

$$\theta^{i}_{est} = \, \arg \max_{\theta } \log \, p(A^{i} |\theta )p(\theta |\mu ,\varSigma ) = \arg \max_{\theta } [\varSigma_{t} \log \, p(a^{i}_{t} |Q_{t} ,\theta )]p(\theta |\mu ,\varSigma )$$

where *A* ^*i*^ represents all the actions by subject *i* and where the dependence of each individual action probability on the parameters *θ* determining the *Q* value was emphasized. Importantly, we set the prior parameters empirically using expectation maximization to find the maximum-likelihood estimates of *μ* and *Σ* given all the data by all the subjects. Based on the parameters *θ* ^*i*^ for each of the subjects, a temporal sequence of PEs was computed for each subject *i* as follows:

$${\text{PE}}^{i}_{t} = \, R^{i} \left( t \right) \, {-} \, Q^{i}_{t - 1} \left( {a_{t} } \right).$$

Thus, rather than doing an individual maximum-likelihood fit, we did a maximum-likelihood fit of the group mean via expectation–maximization and then inferred the posterior parameter estimates for each individual. This reduces variability in the parameter estimates. One alternative in the literature is to assume that all subjects share one and the same parameter. This reduces variability in the regressors, and thus, the SPM beta estimates. The present procedure is what we believe to be a reasonable tradeoff between the two extremes.

### fMRI protocol

fMRI acquisition. Functional imaging was conducted using a 3.0-Tesla GE Signa scanner with an eight channel phase array head coil to acquire gradient echo T2*-weighted echo-planar images as previously described [35, 36]. For each of the two sessions, 310 EPI volumes (~12 min) containing 29 slices were acquired [repetition time (TR) = 2,300 ms, echo time (TE) = 27 ms, matrix size 128 × 128, and a field of view (FOV) 256 × 256 mm^2^, thus yielding an in-plane voxel resolution of 2.7 mm^2^, flip angle *α* = 90 degree). Slices were acquired interleaved with a thickness of 4 mm and no gap. The acquisition plane was tilted 30° from the anterior–posterior commissure. A 3D anatomical image of the entire brain was obtained by using a T1-weighted 3D spoiled-gradient echo pulse sequence (TR = 7.8 ms, TE = 3.2 ms, matrix size 256 × 256, FOV 256 x 256 mm^2^, 1 mm slice thickness, flip angle *α* = 20°, voxel size 1 mm × 1 mm × 1 mm).

fMRI data preprocessing. Functional imaging data were analyzed using SPM8 (Wellcome Department of Imaging Neuroscience, Institute of Neurology, London, UK; http://www.fil.ion.ucl.ac.uk/spm/). After de-noising with ArtRepair (http://cibsr.stanford.edu/tools/ArtRepair/ArtRepair.htm), the following preprocessing steps were performed: acquisition time and motion correction, coregistration of the mean EPI to the anatomical T1 image, spatial normalization and segmentation into tissue classes of the T1 image using the unified segmentation approach as implemented in SPM8 [37] application of the normalization parameters to all functional images, and spatial smoothing with an isotropic Gaussian kernel of 8 mm full width at half maximum (FWHM) kernel.

Small volume correction for multiple comparisons was used within a ventral striatal volume of interest (VOI). We created an fMRI literature-based probabilistic VOI for the VS [13]. We selected 16 recent papers containing data from 325 subjects [5, 6, 35, 38–50]. From each study, the coordinates of PE-related activation for right and the left VS were extracted.

### Statistical analysis

The images were analyzed in an event-related manner using the general linear model approach (GLM) as implemented in SPM8; neuronal activity was modeled using a stick function at the onsets of the feedback. We used a parametric design [32, 51] in which the trial-by-trial PE values from the RW model modulated the amplitude of the trial-related stick. Regressors of interest for the BOLD responses corresponding to the trial-wise PEs were generated by convolving the modulated stimulus functions with the canonical hemodynamic response function (HRF), provided by SPM8. To account for signal fluctuations associated with the movement by susceptibility interaction, the six movement parameters from the realignment preprocessing step were included in the model as additional regressors. The individual contrast images for the contrast of the PE-modulated feedback were then taken to a random-effects group-level analysis using a one sample *t* test. To test for associations with measures of IQ and SLES, these measures were entered as covariates into additional random-effects analyses.

In addition, we performed stepwise multiple regression analyses to test the predictive effect of SLES and PE signaling on fluid IQ using SPSS. These were used to assess (1) the effects of PE signaling and SLES on fluid IQ and (2) tested for interaction effects (moderation) between SLES and PE signaling on fluid IQ by computing an interaction term (specifically, we assigned participants reporting below median SLES a value of one and participants reporting above median SLES a value of two and multiplied this dichotomous variable with the peak VS BOLD PE signal) and added this interaction term to the regression analysis predicting fluid IQ.

To minimize false-positive results due to median split of SLES (and thus reducing effective sample size), we performed an additional stepwise multiple regression analyses entering SLES as a continuous variable into the interaction term after z-transformation (and multiplied this continuous variable with the peak VS BOLD PE signal) and then added this interaction term to the regression analysis.

## Results

On average, participants reported a mean of 16.31 (SD = 4.59) out of 42 SLES. Overall mean fluid IQ was 0.60 (SD = 0.64) after Varimax Rotation (z-transformed). Participants displayed a mean crystallized IQ of 32.31 (SD = 5.67), measured with verbal knowledge test. All subjects made an average of 70.8 ± 6.1 % correct responses and reached criterion (number of reversal stages) on 5.2 ± 1.3 conditions with a learning speed of 0.6 trials. For best-fitting parameter estimates, log likelihoods, and learning rates see Table 1. There was no correlation between fluid IQ and any of the performance parameters (percent correct responses, number of achieved reversal stages, learning speed: all *p* > 0.05). Fluid IQ declined with age (correlation between fluid IQ and age: Pearson’s correlation = −0.61, *p* = 0.013). Fluid IQ was not significantly associated with SLES (Pearson’s correlation = 0.24, *p* = 0.376), and the amount of SLES was not associated with age (Pearson’s correlation = −0.11, *p* = 0.694).

**Table 1:** Best-fitting parameter estimates are shown as median + quartiles across subjects

	*β*_rew_	−*β*_pun_	LL	LR
25th percentile	1.96	0.61	53.15	0.40
Median	2.73	0.84	73.10	0.62
75th percentile	5.03	1.10	109.07	0.77

Also shown are medians and quartiles for the log-likelihood (LL) of the data at the best-fitting parameters and the learning rate (LR). The variables reward/punishment sensitivities (*β*_rew_ and −*β*_pun_) represent the effective reinforcement sensitivity as expressed by the effect of the reinforcement on the subject’s choice behavior. This variable took on value *β*_rew_ if a reward was obtained and −*β*_pun_ if a punishment was obtained

*β*_rew_ reward-sensitivity, −*β*_pun_ punishment-sensitivity, *LL* log-likelihood, *LR* learning rate

### Prediction error signaling in the ventral striatum (VS Bold PE signal)

In the fMRI group of 16 healthy controls, we first observed a significant correlation between the model-free PEs and the BOLD response in the left VS (*x*/*y*/*z* = −18/3/−5, *T* = 3.38, *p*_FWE corrected for VS VOI_ = 0.026). There was also a trendwise correlation in the right VS (*x*/*y*/*z* = 20/6/−5, *T* = 2.47, *p*_FWE corrected for VS VOI_ = 0.057, for whole brain activation see Supplementary Figure 1). Thus, subjects’ VS BOLD activity correlated with the PE learning signal. We term this correlate the VS BOLD PE signal. Below, we examine correlations between this VS BOLD PE signal, SLES, and fluid IQ.

### Correlations between fluid IQ, stressful life events, and VS BOLD PE signal

Fluid IQ was significantly and positively correlated with the VS BOLD PE signal both on the left (*x*/*y*/*z* = −11/8/−8, *T* = 3.89, *p*_FWE corrected for VS VOI_ = 0.012) and on the right (*x*/*y*/*z* = 17/6/−8, *T* = 3.71, *p*_FWE corrected for VS VOI_ = 0.013). Thus, VS functional activation was more closely correlated with model-free habitual PEs in subjects with higher IQ as reported previously [13]. Upon controlling for age by introducing age as an additional covariate into the SPM analysis, VS PE BOLD signal remained associated with fluid IQ (left VS: *x*/*y*/*z* = −16/3/−8, *p*_FWE corrected for VS VOI_ = 0.045, *T* = 3.11, right VS: *x*/*y*/*z* = 20/3/−8, *p*_FWE corrected for VS VOI_ = 0.006, *T* = 4.30), suggesting that this association was not simply explained by an age-related decline in fluid IQ.

**Fig. 2:**
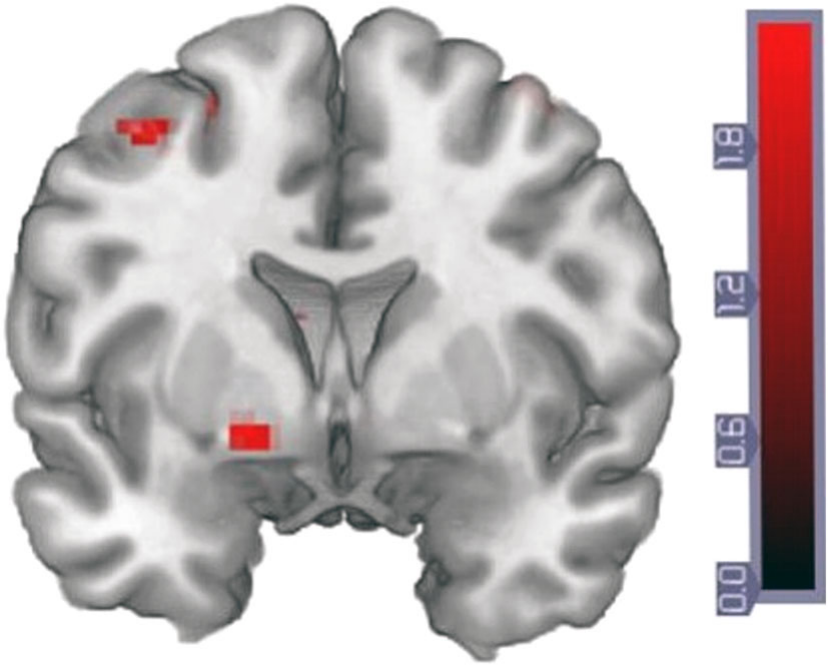
Stressful life events were significantly and positively correlated with the BOLD PE signal in the left VS (*x*/*y*/*z* = −16/3/−8, *T* = 3.02; *p*_FWE corrected for VS VOI_ = 0.047). *Color Scale* represents *T* Values

In addition, SLES was significantly and positively correlated with the BOLD PE signal in the left VS (left VS: *x*/*y*/*z* = −16/3/−8, *T* = 3.02, *p*_FWE corrected for VS VOI_ = 0.047 (Fig. 2), though not in the right VS (*p* > 0.2). This association remained significant when entering age as an additional covariate into the SPM analyses (left VS: *x*/*y*/*z* = −16/3/−8, *p*_FWE corrected for VS VOI_ = 0.048, *T* = 3.10). As stated above, fluid IQ was not significantly associated with SLES (Pearson’s correlation = 0.24, *p* = 0.376).

### Effects of stressful life events and VS BOLD PE signal on fluid IQ

Stress has been shown to increase the habitual, or model-free, component of behavior [52]. The PE regressor we have used is one such model-free, habitual, learning signal [8]. We were thus interested to know whether stress increases the contribution of this basic learning signal to the more general index of flexible learning measured by fluid IQ. We therefore performed a stepwise regression analysis, asking whether the product of SLES and left VS BOLD PE signal explains additional variance in the fluid IQ measure beyond the effects of SLES and VS BOLD PE signal alone.

**Fig. 3:**
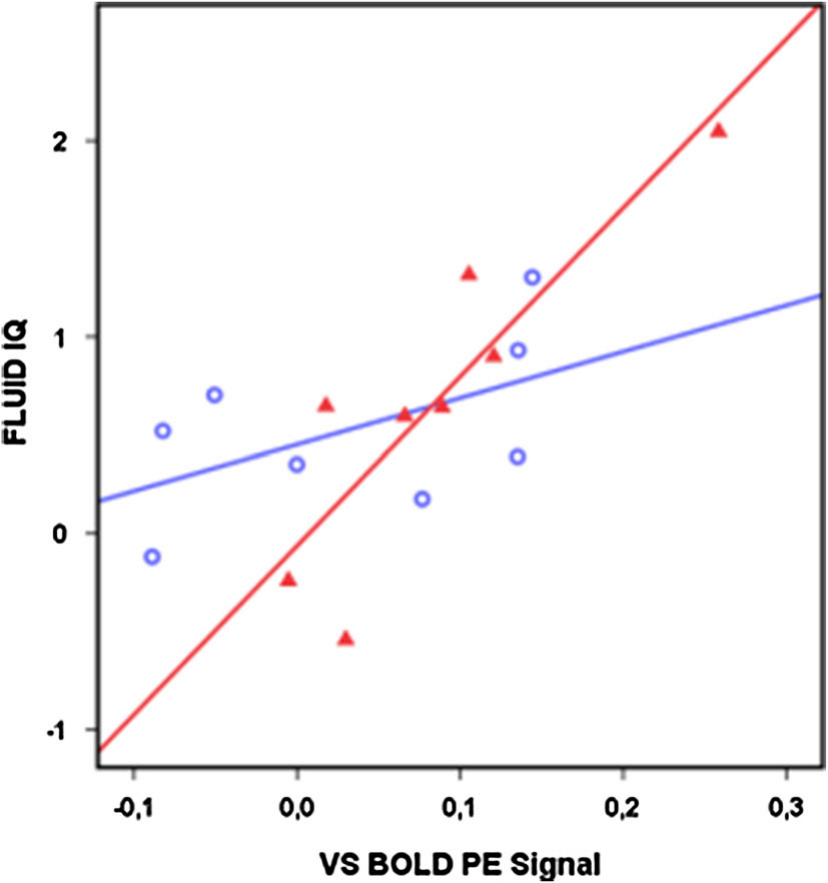
Effects of stressful life events and VS BOLD PE signal on fluid IQ. Subjects reporting stressful life events above the median are depicted in red solid triangles (*R* ^2^ = 0.759). Subjects reporting below the median in blue transparent circles (*R* ^2^ = 0.287). The interaction term is significant (*β* = 1.19, *T* = 2.53, *p* < 0.05), indicating that in subjects reporting more stressful life events, the VS BOLD PE signal correlates more strongly with fluid IQ. An outlier analysis was performed for each included data point (*n* = 16) revealing no relevant leverage effects (*z* value < 3, Cook’s distance < 1, max = 0.218, SD = 0.061)

The VS BOLD PE signal again correlated significantly with fluid IQ (*β* = 0.89, *T* = 3.61, *p* < 0.005), while SLES did not correlate with fluid IQ (*p* > 0.20). Thus, in our sample, fluid IQ and SLES were not directly correlated. However, there was a significant effect of the interaction of VS BOLD PE signal x SLES (*β*=1.19, *T*=2.53, *p*<0.05). This interaction term increased the fraction of variance in fluid IQ that was explained from 53 to 69 %—a significant change (*F*_Change_ = 6.39, *p* < 0.05). In order to understand the direction of this effect, we plotted fluid IQ as a function of VS BOLD PE signals using a median split on SLES (above or below 17 reported events). Figure 3 shows that the association between VS PE BOLD signaling and fluid IQ was increased in subjects reporting more SLES (*R* ^2^ =0.76). This interaction term remained significant when adding SLES as a continuous variable (*ß* = 0.43, *T* = 2.53, *p* < 0.05). We performed an outlier analysis for each included data point (*n* = 16) revealing no relevant leverage effects (*z* value<3, Cook’s distance<1, max=0.218, SD=0.061).

## Conclusion

We learn from making mistakes and need to adapt our predictions in the face of changing circumstances; iterative learning via PEs plays a major role in such learning processes [7, 53]. The present results are the first reported data showing that a basic neurobiological learning signal—the ventral striatal PE signal—and SLES interact to predict individual differences in fluid intelligence, even when correcting for the decline of fluid IQ with age. Several points merit comment.

First, it may seem surprising that the relationship of SLES to the BOLD PE signal is in the same, positive, direction as that of fluid IQ. However, stress is well known to promote habitual, model-free responses in humans and animals. The VS BOLD PE signal quantifies how closely the BOLD signal in the VS correlated with a standard model-free learning signal. This is a measure of the co-alignment of the two time-varying signals, not of the magnitude of either of them. As such, a stronger VS BOLD PE signal is in keeping with more habitual, model-free learning. On the other hand, fluid IQ among other cognitive attributes captures planning ability and other goal-directed and more challenging (model-based) cognitive functions [54]. The fact that higher fluid IQ is positively associated with a stronger alignment of VS activity with the model-free (habitual) learning signal is thus, at first sight, a counterintuitive finding. However, the model-free learning signal may be correlated with more complex model-based signals in this particular task, and there is evidence that a VS PE signal may in fact comprise both model-free and model-based components [55]. One possible explanation is that this alignment is an expression of the engagement of several different learning strategies in parallel (including goal-directed—model-based and habitual—model-free), which is in accordance with our previous interpretation of study results. We had observed that fluid IQ is associated with the VS BOLD PE signal, a putative signature of habitual (model-free) learning even when controlling for behavioral fit, which indicates how strongly the observed behavior is accounted for by PE-driven learning [13]. This issue needs re-visiting in the light of the current data, as here, subjects who had not experienced much life stress showed only a small correlation between IQ and VS BOLD PE signal. Thus, our finding of a correlate between fluid IQ and VS BOLD PE signal is driven by those subjects who have experienced substantial life stress, which may have gradually shifted their flexible cognitive capacities toward more model-free strategies.

Our results complement previous studies on the effect of (acute) stress on dopamine signaling and ventral striatal PE encoding. Stress exposure facilitates ventral striatal dopamine release [22, 23, 56], and Robinson et al. [14] recently observed increased ventral striatal PE signaling (model-free) of negative errors of reward prediction (i.e. when received outcome is smaller than expected) during acute stress. Additionally, Otto et al. [16] reported that acute stress exposure reduces the amount of model-based learning in subjects with low working memory capacity (a measure closely related to fluid intelligence and general cognitive ability), while model-free learning was unaffected and thus shifts the balance from goal-directed (model-based) toward more habitual (model-free) learning strategies during decision making. We now show that self-reported SLES, which reflect chronic rather than acute stressors, are positively associated with ventral striatal BOLD PE signal and that SLES interact with the neuronal learning signature to predict fluid IQ. Thus, it seems feasible that chronic stressors have an effect on VS PE signaling in that they induce a stronger encoding of the PE signal during habitual learning, effects which interact to predict individual differences in fluid IQ—above and beyond the (negative) effect of age on fluid IQ.

Despite the link between stress and the development of various psychopathologies [57, 58], few studies have compared the neurophysiological effects of acute and repeated stressors. There is ample evidence to indicate both types of stress influence the dopaminergic system, pointing in the direction of differential effects of acute and chronic stress [14, 16]. These modulations of dopaminergic firing have been seen in the ventral tegmental area and the VS [22, 23, 56] core areas for the neuronal representation of reward PEs, reward anticipation, learning from reinforcement [59, 60], and flexible behavioral adaptation (fluid IQ). Furthermore, studies suggest that genetic variation in the dopamine system moderates the effects of acute and chronic stress [58, 61] and are associated with individual differences in working memory capacity and other aspects of fluid intelligence [62–65].

Our data indicate that the above reported VS BOLD PE signal works as an indirect neuronal signature of stress experience that is in part driven by a dopaminergic modulation which might shift learning strategies in highly flexible subjects (with high levels of fluid IQ) towards more habitual rather than goal-directed learning strategies. These data might allow for the speculation that it may be ecologically salient to encode errors of reward prediction more strongly when life experiences are mainly adverse and thus reduce cognitive demands in complex and threatening situations.

An important limitation to our findings is that the results are based on a small sample and therefore will require replication. Nevertheless, the direction of the stress effects is consistent with a recent report of the impact of acute stress on PE signals in a reversal learning task [14]. Also, recent evidence [24] points toward the direction of gender differences in reward-related decision processing under stress. Therefore, our study will have to be repeated in an independent female sample. The findings are correlational, and hence, no statements about causality can be made. The various directional interpretations are, however, worth disentangling. It appears that stress alters how IQ relates to the VS PEs. The importance of the findings derive from the fact that this is the first study showing that an association between reward PE signaling and intelligence is moderated by life stress experience and this suggests stressful life experiences may sensitize the dopaminergic system toward more habitual decision making.

## Electronic supplementary material

Below is the link to the electronic supplementary material.

Supplementary Figure 1: Striatal activation at p <0.001 whole brain activation (left striatum x/y/z = -26/1/-8, p<0.001, F=27.91, cluster size (voxel) =17; right striatum: x/y/z = 25/6/-3, p<0.001, F=23.74, cluster size (voxel) = 18). (TIFF 298 kb)
