# Supplementary figures and images for: The effects of life stress and neural learning signals on fluid intelligence
Source: Eur Arch Psychiatry Clin Neurosci. 2014 Aug 21;265(1):35–43. doi: 10.1007/s00406-014-0519-3 (PMC4311068; doi:10.1007/s00406-014-0519-3)

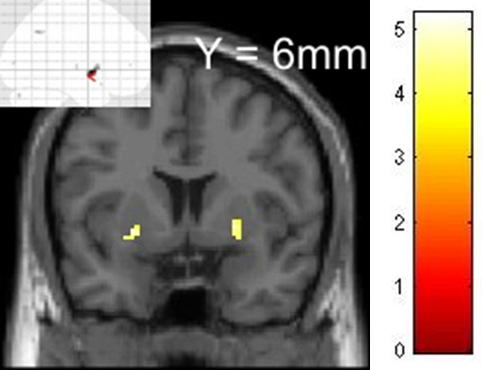

Supplement: Supplementary file 1 — Supplementary Figure 1: Striatal activation at p <0.001 whole brain activation (left striatum x/y/z = -26/1/-8, p<0.001, F=27.91, cluster size (voxel) =17; right striatum: x/y/z = 25/6/-3, p<0.001, F=23.74, cluster size (voxel) = 18). (TIFF 298 kb) [file 406_2014_519_MOESM1_ESM.tif]
